# Editorial: Cognitive and Motor Control Based on Brain-Computer Interfaces for Improving the Health and Well-Being in Older Age

**DOI:** 10.3389/fnhum.2022.881922

**Published:** 2022-04-06

**Authors:** Abdelkader Nasreddine Belkacem, Tiago H. Falk, Takufumi Yanagisawa, Christoph Guger

**Affiliations:** ^1^Department of Computer and Network Engineering, College of Information Technology, United Arab Emirates University, Al Ain, United Arab Emirates; ^2^Institut National de la Recherche Scientifique, University of Québec, Montréal, QC, Canada; ^3^Department of Neurosurgery, Graduate School of Medicine Osaka University, Suita, Japan; ^4^Center for Information and Neural Networks, National Institute of Information and Communications Technology, Suita, Japan; ^5^Institute for Advanced Co-Creation Studies, Osaka University, Suita, Japan; ^6^g.tec Medical Engineering GmbH, Schiedlberg, Austria

**Keywords:** brain computer interface, brain aging, BCI (brain control interface), EEG, stroke, Alzheimer, post-stroke

This editorial summarizes the contributions to the Frontiers Research Topic “*Cognitive and Motor Control Based on Brain-Computer Interfaces for Improving the Health and Well-Being in Older Age*,” established under the Frontiers in Human Neuroscience (section: Brain-Computer Interfaces), Frontiers in Neuroergonomics (section: Consumer Neuroergonomics), and Frontiers in Neuroscience (section: Neural Technology) journals.

Everyone is subject to the aging process and to experiences associated with physical and health changes, which include but are not limited to changes in memory and brain function. These changes may be debilitating due to increased dependence of people as they get older. Many engineering tools and neuroscience breakthroughs have been used to assist these individuals in their daily life activities or to enhance, restore, and improve the effects of the many age-related changes.

Emerging technologies such as cognitive and motor control based on invasive and non-invasive brain-computer interfaces (BCIs) provide opportunities to improve the health and wellbeing of the elderly. These external approaches may assist elderly patients improve motor function (e.g., in stroke survivors) and induce and/or facilitate neuroplastic changes associated with motor rehabilitation, caretakers and family members to better communicate with these patients, and healthcare professionals to better monitor and detect changes in the patients' health status. While there has been great progress in the field of BCI over the last decades, the development of hardware and software solutions for home-based BCI applications that can be used by individuals with reduced technical oversight is still lacking. In addition, user-friendly, wearable, portable, and wireless BCI applications, as well as more longitudinal neuroscience studies are essential to understand how BCI applications can improve the quality of life of elderly people.

This editorial paper will introduce the five papers appearing in this Research Topic. The papers touch on different aspects of BCI usage for improving the health and wellbeing in older age. As will be summarized, several innovations in bio-signal processing methods and decoding algorithms are introduced that ease the effect of aging on the human brain and enrich the mutual interplay between senior citizens and machines. With the objective of assisting in cognitive support, physical support, and rehabilitation, neurofeedback tools and novel control applications for neural prostheses, smart home, wheelchair, and exoskeleton technologies are also introduced. Lastly, being able to detect neurodegenerative diseases, such as dementia and Alzheimer's disease (AD), at early stages and tracking their severity levels can be important for clinicians, thus providing them with additional insight on disease progression. New multimodal tools and signal processing techniques are described herein to improve AD severity level monitoring.

It is widely known that stroke is a leading cause of acquired long-term upper extremity motor disability. Therefore, a study of crossover-controlled trial NCT02098265 has focused on improving quality of life of stroke survivors with affected motor cortices disregarding stroke severity and chronicity. In the paper “*Ipsilesional Mu Rhythm Desynchronization (ERD) Correlates with Improvements in Affected Hand Grip Strength and Functional Connectivity (iCoh) in Sensorimotor Cortices Following BCI-FES Intervention for Upper Extremity in Stroke Survivors,”* Remsik et al. examine whether the ipsilesional Mu rhythm desynchronization of the cerebral cortical sensorimotor areas [Brodmann's areas (BA) 1–7] is localized and tracks with changes in grip force strength during movements of the affected hand compared to rest. The authors tested the hypothesis that BCI interventions can result in changes in frequency-specific directional flow of information transmission (direct path functional connectivity) in BA 1–7 by measuring changes in the isolated effective coherence (iCoh) between cerebral cortical sensorimotor areas thought to relate to electrophysiological signatures of motor actions and motor learning. Results show that the ipsilesional Mu rhythm desynchronization is correlated with improvements in the grip strength of the affected hand and functional connectivity in sensorimotor cortices following the BCI-FES (functional electrical stimulation) intervention for upper extremity in stroke survivors.

BCI technologies have increasingly been shown to be promising tools to be used during rehabilitation, facilitating motor recovery after stroke. However, we still have limited understanding of the changes in functional connectivity and behavioral outcomes associated with their use. In the paper “*BCI Training with FES: Facilitating Changes in Interhemispheric Functional Connectivity and Motor Outcomes Post-Stroke,”* Sinha et al. studied the effects of an electroencephalography (EEG)-based BCI intervention with functional electrical stimulation (FES) on the resting-state functional connectivity (rsFC) and motor outcomes in the stroke recovery of 23 post-stroke patients with upper limb motor impairments. Results showed that the BCI intervention with FES can facilitate interhemispheric connectivity changes and upper limb motor recovery in patients after stroke.

BCIs have also emerged recently as a useful tool to help clinicians detect and characterize neurodegenerative disorders, such as Alzheimer's disease (AD). In the paper “*Multimodal Prediction of Alzheimer's Disease Severity Level Based on Resting-State EEG and Structural MRI*,” Jesus et al. proposed a multimodal prediction algorithm to detect the severity level of patients diagnosed with AD. In particular, several features from EEG and structural magnetic resonance imaging (MRI) scans were extracted and combined with different feature selection methods, machine learning algorithms, cross-modality fusion schemes. Results showed the importance of a multimodal EEG-MRI system for practical applications that monitor AD progression and showed the complementarity of the two neuroimaging modalities for the task at hand.

Brain-computer interfaces have also seen applications in neurofeedback protocols to assist patients with different disorders, including depression and anxiety. In paper “*EEG-based neurofeedback with network components extraction: a data-driven approach by multilayer ICA extension and simultaneous EEG-fMRI measurements*,” Ogawa et al. propose a multilayer independent component analysis extension and simultaneous EEG-fMRI measurement for EEG-based neurofeedback scheme based on extracting network components. A new stacked pooling and linear components estimation (SPLICE) method was proposed as a multilayer extension of independent component analysis and independent subspace analysis. Results suggested the proposed method is a promising alternative and a practical signal processing technique to extract functional connectivity related features from EEG signals. A real-time EEG neurofeedback system was described based on SPLICE and shown to improve depressive symptoms. In addition, possible ways to modify their neurofeedback protocol including experimental design, sample size, and online processing were also discussed.

Lastly, conventional BCI methods typically rely on mental imagery tasks, such as motor imagery, or on audio-visual reactive paradigms, such as the P300 evoked potential. In the paper “*A Novel P300 Paradigm for Brain-Computer Interface Based on Simple-Commanded Electric and Vibration Tactile Stimulation*,” Chu et al. propose a novel tactile-stimuli P300 paradigm for BCI, targeted at individuals with reduced learning ability or difficulty in maintaining attention. Both electrical and vibration stimuli were investigated and promising results were achieved with both methods; an average accuracy of 94.88% was achieved for electrical stimuli and 95.21% for vibration stimuli. Such novel BCI paradigm may show useful within an elderly population in which focus and learning abilities may be compromised due to aging and/or disease.

## Author Contributions

All authors listed have made a substantial, direct, and intellectual contribution to the work and approved it for publication.

## Funding

AB was supported by the ASPIRE Young Investigator Award (AYIA 2020), funded by the ASPIRE under contracts 21T057-AYIA20-002 and by the United Arab Emirates University (Grant No. G00003270 31T130). TF was supported by the Canada Natural Sciences and Engineering Research Council (NSERC, RGPIN-2021-03246).

## Conflict of Interest

CG is the CEO of g.tec medical engineering GmbH. The remaining authors declare that the research was conducted in the absence of any commercial or financial relationships that could be construed as a potential conflict of interest.

## Publisher's Note

All claims expressed in this article are solely those of the authors and do not necessarily represent those of their affiliated organizations, or those of the publisher, the editors and the reviewers. Any product that may be evaluated in this article, or claim that may be made by its manufacturer, is not guaranteed or endorsed by the publisher.

